# Rapid identification of *Amanita citrinoannulata* poisoning using colorimetric and real-time fluorescence and loop-mediated isothermal amplification (LAMP) based on the nuclear ITS region

**DOI:** 10.1016/j.fochms.2022.100082

**Published:** 2022-02-04

**Authors:** Jie Gao, Ruibin Xie, Nan Wang, Juan Zhang, Xiaoyun Sun, Hongjing Wang, Jianxin Tan, Ailiang Chen

**Affiliations:** a Institute of Quality Standard & Testing Technology for Agro-Products, Key Laboratory of Agro-product Quality and Safety, Chinese Academy of Agricultural Sciences, Beijing 100081, China; b College of Food Science and Technology, Hebei Agricultural University, Baoding 071001, China

**Keywords:** ITS region, Loop-mediated isothermal amplification (LAMP), Authentication, *Amanita citrinoannulata*

## Abstract

Health concerns and financial losses caused by mushroom poisoning have been reported worldwide. *Amanita citrinoannulata*, a poisonous mushroom commonly found in China, results in a toxic reaction in humans after mistaken ingestion. To reduce the mistaken ingestion of poisonous mushrooms and to improve clinical diagnosis of mushroom poisoning, a rapid mushroom species identification method is required. Such identification methods could be advantageous in the identification of other poisonous mushroom species. This study developed two rapid and sensitive methods for the detection of *A. citrinoannulata* utilizing colorimetric and real-time loop-mediated isothermal amplification (LAMP) technology and specifically designed primers for the internal transcribed spacer (ITS) genes of *A. citrinoannulata*. The methods demonstrated high sensitivity as 0.2 ng of *A. citrinoannulata* DNA could be detected, with no cross-reaction with 41 non-target mushroom species. The entire detection process could be completed within 40 min without requiring complex instruments and can be observed by the naked eye. Therefore, these novel methods can be used for the identification of fresh and cooked mushroom samples and vomit samples, which contain only 1% *A. citrinoannulata*. Furthermore, these methods facilitate the detection of mushroom poisoning, and thus, have potential to reduce the number of mushroom poisoning-related deaths worldwide.

## Highlights

• New colorimetric and real-time fluorescence LAMP for A. citrinoannulata detection was developed.
• The method is specific, sensitive and could be finished within 30 min.
• Identification of poisonous mushrooms in processed or digested samples was achieved.
• The method could be used for on-site detection without needing complicated instruments.

## 1 Introduction

As a delicious wild food, mushrooms are loved and collected by many people from wild fields. However, many mushrooms are poisonous and could induce serious illness. Such mushrooms are difficult to identify due their similarity in appearance with edible mushrooms. Globally, reports of the number of mushroom poisoning-related deaths have been increasing every year (Cervellin et al., 2018, Gold et al., 2021, Li et al., 2021), distressing healthcare professionals. In China, of the 13 307 known pathogenic outbreaks reported in 2003–2017, 31.8% were caused by poisonous mushrooms (Li et al., 2020). With the increasing number of related deaths, mushroom poisoning poses a serious food safety threat worldwide.

In 2019, *Amanita citrinoannulata,* a toxic mushroom, caused a food poisoning outbreak in China; however, its toxicity had not been previously recorded. *A. citrinoannulata* was described in China in 2018 and was found to be distributed in the Chongqing, Yunnan, Jiangsu, Shandong, and Guizhou provinces (Cui et al., 2018). *A. citrinoannulata* is a light gray or brown mushroom that is easily perceived as nontoxic. The consumption of poisonous mushrooms, such as *A. citrinoannulata,* leads to high medical costs (Govorushko et al., 2019). Therefore, establishing a rapid, field-portable, and simple detection method for identifying poisonous mushrooms is of great significance in source investigation, clinical diagnosis, and correct treatment.

Professional mycologists currently identify mushroom varieties by extensively evaluating the morphological characteristics of mushrooms. However, the morphology of a particular mushroom can vary greatly across different locations owing to variations in environmental and genetic factors. The high degree of variability in mushroom morphology has greatly challenged the accurate identification of mushrooms. For instance, mushroom species were accurately identified in only 43 % of the 51 cases of mushroom poisoning treated in University Hospital Bern in Switzerland (Keller et al., 2018). Therefore, the development of improved identification methods for poisonous mushroom species is of great significance. Scientists have developed many identification methods for poisonous mushrooms, including immunoassays, which have high sensitivity, but take a long time and hence, are not compatible to be used in clinical settings. Additionally, spectroscopic analysis, liquid chromatography-tandem mass spectrometry, and ultraperformance liquid chromatography tandem mass spectrometry (Gicquel et al., 2014, Pyrzyñska and Trojanowicz, 1999, Zhang et al., 2016) are highly sensitive and fast in species detection, but they require expensive equipment and extensive sample pretreatment, which are sometimes difficult to execute in remote areas and resource-limited settings. Moreover, in mushroom poisoning cases, mushroom samples are not usually well preserved, especially cooked mushroom samples or cases of vomiting. In recent years, nucleic acid-based detection methods have been developed using the specificity of target genes and have achieved good stability even in deep-processed samples. PCR is one of the most commonly used nucleic acid detection methods. However, the complex heating and cooling process of PCR requires approximately two hours for PCR detection. Developing more rapid detection methods is necessary for the initial treatment and research of mushroom poisoning.

Loop-mediated isothermal amplification (LAMP) is a technique for DNA amplification under isothermal conditions. This technology has been widely used in the molecular detection and identification of pathogenic fungi in medicinal, food, and agricultural products (Leonardo et al., 2021, Moehling et al., 2021, Mori and Notomi, 2009). More recently, it has also been utilized for the diagnosis of the SARS-CoV-2 coronavirus (Chaouch, 2021). At present, LAMP technology has been used for the identification of *Amanita*. For example, Vaagt et al. (2013) established a detection method for *Amanita phalloides*, and the bands were detected using agarose gel. More recently, He et al. (2019) established different detection methods for fatal *Amanita* based on the principle of using hydroxynaphtol blue (HNB) dye and detecting color changes.

In this study, we aimed to develop a rapid, sensitive, specific, and naked-eye LAMP detection method for *A. citrinoannulata* using a useful genetic marker, the internal transcribed spacer (ITS) region of the ribosomal DNA (Ratnasingham & Hebert, 2007). ITS was proposed as a common barcode region for fungal identification (Schoch et al., 2012). Therefore, LAMP primers were designed using the ITS sequence of *A. citrinoannulata*, and a visualization and real-time LAMP detection method was established. In addition, considering the practical application scenarios of the method, mushrooms were treated with boiling water and digestion of mushrooms by gastric juice were simulated. The LAMP detection method of *A. citrinoannulata* developed in this study may be of great significance for the prevention and diagnosis of poisoning and rapid identification of mushroom species in poisoning incidents. To the best of our knowledge, this is the first established detection method for *A. citrinoannulata*.

## 2 Materials and methods

### 2.1 Sample collection

To determine the specificity of the colorimetric LAMP assay established in this study, fresh samples of 42 different mushroom species were used (Table 1). *Hypsizygus marmoreus*, *Flammulina filiformis*, *Lentinula edodes*, and *Pleurotus eryngii* samples were purchased from supermarkets; *Chlorophyllum molybdites* was provided by the Institute for Agri-food Standards and Testing Technology, Shanghai Academy of Agricultural Sciences, China. Other mushroom species were collected in Lijiang, Yunnan, China from July to September 2020. All mushroom species were preliminarily distinguished by morphological identification and then further identified by Sanger sequencing based DNA barcoding method described in 2.2. All samples were stored at −80℃ for subsequent experiments.

**Table 1:** Mushroom samples used in this study.

Family	Species Name	Poisonous/ Edible	Genbank Accession Number
*Amanitaceae*	*Amanita citrinoannulata*	Edible	MW192480
	*Amanita concentrica*	Poisonous	MW192487
	*Amanita griseofolia*	Poisonous	MW192459
	*Amanita hemibapha*	Edible	MW192463
	*Amanita parvipantherina*	Poisonous	MW192484
	*Amanita pseudovaginata*	Poisonous	MW192492
	*Amanita sepiacea*	Poisonous	MW192488
	*Amanita spissacea*	Poisonous	MW192473
*Agaricaceae*	*Chlorophyllum molybdites*	Poisonous	MW192451
	*Leucoagaricus rubrotinctus*	Poisonous	MW192455
	*Macrolepiota dolichaula*	Edible	MW192456
*Boletaceae*	*Boletus kauffmanii*	Edible	MW192464
	*Butyriboletus yicibus*	Edible	MW192467
	*Suillus bovinus*	Edible	MW192452
	*Tylopilus neofelleus*	Poisonous	MW192465
	*Tylopilus microsporus*	Poisonous	MW192490
*Cortinariaceae*	*Hebeloma crustuliniforme*	Poisonous	MW192482
*Hydnaceae*	*Hydnellum caeruleum*	Edible	MW192469
	*Hydnellum concrescens*	Edible	MW192472
*Inocybaceae*	*Inocybe mixtilis*	Edible	MW192471
	*Inocybe rimosa*	Poisonous	MW192491
*Lyophyllaceac*	*Hypsizygus marmoreus*	Edible	MW192478
*Marasmiaceae*	*Gymnopus subnudus*	Edible	MW192453
*Pleurotaceae*	*Pleurotus eryngii*	Edible	MW192475
	*Pleurotus ostreatus*	Edible	MW192489
	*Pleurotus ostreatus*	Edible	MW192494
*Psathyrellaceae*	*Panaeolus subbalteatus*	Poisonous	MW192454
*Russulaceae*	*Lactarius subbrevipes*	Edible	MW192457
	*Russula crustosa*	Edible	MW192481
	*Russula rosacea*	Edible	MW192466
	*Russula sanguinea*	Edible	MW192493
	*Russula senecis*	Edible	MW192462
	*Russula variata*	Edible	MW192461
	*Russula velenovskyi*	Edible	MW192468
*Tricholomataceae*	*Laccaria aurantia*	Edible	MW192458
	*Flammulina filiformis*	Edible	MW192476
	*Lentinus edodes*	Edible	MW192477
	*Rhizocybe alba*	Edible	MW192460
	*Tricholoma imbricatum*	Poisonous	MW192474
	*Tricholoma matsutake*	Poisonous	MW192486
	*Tricholoma imbricatum*	Poisonous	MW192483
	*Tricholoma saponaceum*	Poisonous	MW192470

### 2.2 DNA extraction, PCR amplification, and Sanger sequencing

Mushrooms fruiting body samples were placed into a mortar and liquid nitrogen was added quickly to freeze-dry the mushrooms, which were then ground into powder with a pestle. Subsequently, 100 mg powder of each mushroom sample was taken for DNA extraction. The DNA from the powder was extracted using the New Genome DNA extraction Kit of plant tissues (Tiangen, Beijing, China) according to the manufacturer’s instructions. Total DNA was stored at −20 °C until further analysis. The concentration of DNA was determined using a Nanodrop 2000 ultramicro-spectrophotometer (Thermo Fisher Scientific, Waltham, United States), with OD values read at 260 nm and 280 nm. DNA purity was determined using the 260/280 nm ratio; the OD value was 1.8–2.2, which revealed a good sample quality that could meet the requirements of subsequent experiments.

A 25 μL PCR mixture, containing a total volume of 2 × Taq PCR Master Mix, 3 μL of each primer, 4.5 μL of ddH_2_O, and 2 μL of DNA was used. Universal primers ITS4 (5′-TCCTCCGCTTATTGATATGC-3′) and ITS5 (5′-GGAAGTAAAAGTCGTAACAAGG-3′) were used to amplify the DNA of all samples using a PCR thermal cycler (Applied Biosystems, California, United States). The amplification was performed under the following conditions: 94 °C initial denaturation for 5 min; followed by 30 cycles of denaturation at 94 °C for 30 s, primer annealing at 58 °C for 30 s, and extension at 72 °C for 30 s; and a 72 °C final extension for 10 min. The amplified products were evaluated using 1.5% agarose gel electrophoresis and sent to Beijing Sangon Biotechnology Technology (China) for Sanger sequencing. The sequences were analyzed using the National Center for Biotechnology Information (NCBI) basic local alignment search tool (BLAST) and also compared with published sequences to confirm the mushroom species. Finally, the obtained sequence was uploaded to GenBank database (accession number in Table 1) (Wang et al., 2021).

### 2.3 Primer design for species-specific primers

**Fig. 1:**
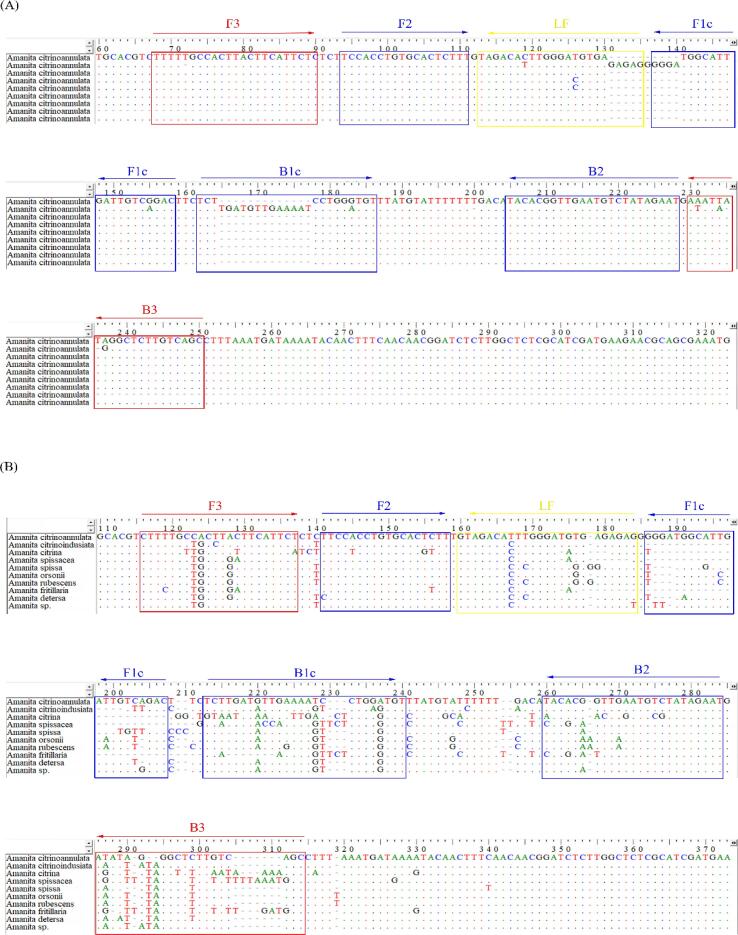
Positions of the new primer sets designed for the LAMP amplification of the ITS gene fragments. (A) LAMP primers covered nine different ITS sequences of *A. citrinoannulata*, among which only a very small mismatch base. (B) Mismatch between *A. citrinoannulata* and nontarget poison mushrooms species at primer binding sites.

The available ITS gene sequences of *A. citrinoannulata* (accession numbers MH508316.1) were downloaded from the GenBank database (https://www.ncbi.nlm.nih.gov/nuccore) to confirm the conserved regions of *A. citrinoannulata.* Primer binding sites were selected to ensure coverage of intraspecific conserved regions. We selected the species with the same branch or a very similar shape as *A. citrinoannulata* in phylogenetic analysis. Although we did not collect all the species, we used their sequences for sequence alignment. In addition, using the BioEdit version 7.0.9, alignment was performed between the *A. citrinoannulata* ITS gene sequence and ITS sequences from species closely related to *A. citrinoannulata*, including *A. citrinoannulata* (MH508316.1), *A. citrinoindusiata* (MH508320.1), *A. citrina* (MH508311.1), *A. spissacea* (AB015683.1), *A. spissa* (MT863751.1), *A. orsoni* (MW425331.1), *A. rubescens* (MH508553.1), *A. fritillaria* (MH508366.1), *A. detersa* (MH508328.1), and *Amanita* sp.(MN647008.1). We manually selected the primer binding sites to ensure that *A. citrinoannulata* had sufficient mismatch with related species in the same branch. Mismatch between *A. citrinoannulata* and related non-target *Amanita* species in the region of the designed primers is shown in Fig. 1.

The LAMP primer set that was used in this study was designed using the GLAPD website (http://cgm.sjtu.edu.cn/GLAPD/online/group_specific.html) based on the ITS gene sequence of *A. citrinoannulata*. Fig. 1 also shows the region of primer design and the F3 and B3c DNA regions which were used to design the inner primers (F3 and B3); F1 and F2 were used to design the internal primers (FIP); and the loop primer (LF) was designed using the LFc region. The primers (BIP) were designed using the B1c and B2c regions. The theoretical specificity of the new primers was verified using Primer-BLAST in NCBI. The primers were synthesized by Sangon Biotech (Shanghai, China).

### 2.4 Colorimetric LAMP procedure and Real-time LAMP procedure

The colorimetric LAMP reaction was carried out in a total volume of 10 μL. Specifically, primers were mixed in advance according to a volume ratio of F3 (10 μM): B3 (10 μM): LF (10 μM): FIP (10 μM): BIP (10 μM) = 1:1:2:8:8. In the reaction mixture, 2 µL of sample DNA (10 ng/μL) was used as the template. The mixture also contained 5 μL WarmStart® Colorimetric LAMP 2X Master Mix (New England Biolabs, Massachusetts, United States), 2.2 μL mixed primers, diluted by adding 0.6 μL water to fill. The colorimetric LAMP reaction mixture was incubated at 65 °C for 25 min. The reaction was stopped by setting the temperature at −20 °C for 1 min, and the results were then observed. It is to be noted that positive results change color from pink to yellow, while negative results stay in pink, and the color contrast remains very strong.

The real-time LAMP reaction was performed using the Applied Biosystems (ABI) 7500 Fast Real-time PCR System (Applied Biosystems, California, United States) to amplify the real-time LAMP mixture. The LAMP mixture contained 5 μL of the WarmStart LAMP 2X Master Mix (New England Biolabs, Massachusetts, United States), 2.2 μL mixed primers, 0.2 μL LAMP Fluorescent Dye (50X) (New England Biolabs, Massachusetts, United States), and 2 μL of DNA (10 ng/μL), and ddH_2_O was added to a final volume of 10 μL. The reaction program was set to 65 °C, 1 min per cycle, for a total of 90 cycles. In this way, the threshold cycle (C_t_) was set to be equal to the fastest time for the product to reach the detectable threshold of the fluorescence signal. Based on these two mixtures and program settings, we performed subsequent specificity experiments, sensitivity experiments, and applicability experiments.

### 2.5 Specificity and sensitivity analysis

To verify the specificity of the LAMP primers, colorimetric and real-time LAMP were applied to all DNA samples. If only the color of the positive control (*A. citrinoannulata*) changed from pink to yellow, whereas the pink color of the negative and blank controls (Other species in Table 1 except positive control and ddH_2_O) remained unchanged, the specificity of the primer group was strong. On a specificity analysis basis, we carried out the sensitivity experiment. The experiment was performed with different concentrations of DNA from *A. citrinoannulata* with dilutions ranging from 100 ng/μL to 0.1 pg/μL (1:10 dilution series) with ddH_2_O, and the sensitivity range was observed.

### 2.6 LAMP method applicability analysis

In order to further verify the applicability of the LAMP method established in this study, we simulated the processes of mushroom processing and body digestion. A series of treated samples were analyzed by LAMP. We used a mixture of three types of mushroom species, including a common poisonous mushroom (*Chlorophyllum molybdites*), common edible mushroom (*Lentinula edodes*), and positive control (*A. citrinoannulata*), mixed at different ratios (*Chlorophyllum molybdites*: *Lentinula edodes*: *A. citrinoannulata* = 1:1:98, 25:25:50, or 54:45:1).

Two groups were established for different treatments. One group was treated with boiling water for 15 min to demonstrate that the colorimetric method could be used for cooked mushroom samples. In the other group, a cooked mushroom mixture was incubated with 1 mL artificial gastric juice for 3 h at a constant temperature and a shaking table was used to simulate the process of human digestion of mushrooms. The in vitro simulated digestion in artificial gastric juice was conducted according to the methods of Pan et al., 2019, Hodgkinson et al., 2019. Finally, DNA was extracted from both groups of mushroom mixtures for LAMP analysis.

## 3 Results

### 3.1 Primer sequences

The five primers synthesized for this experiment were designed to be highly specific to the target sequence. These designed primers showed improved specificity and provided enhanced DNA amplification performance. When the content of the amplified sequences reached a certain limit, the color of the reaction mixture changed from pink to yellow, allowing direct visual observation of the colorimetric results. The primers lacked a primer dimer and met specific requirements with respect to the GC content and annealing temperature, as indicated in Table 2.

**Table 2:** LAMP primer sequences used in this study.

Primer name	Sequence (5′ → 3′)	Tm (°C)	GC content (%)	Length
F3	CTTTTGCCACTTACTTCATTCT	53.9	36.4	22
B3	GCTGACAAGAGCCCTATAT	49.1	47.4	19
FIP	GTCTGACAATCAATGCCATCCCTTCCACCTGTGCACTCTT	83.7	50.5	40
BIP	TCTTGATGTTGAAAATCCTGGATGTATTCTATAGACATTCAACCGTGTA	79.9	34.7	49
LF	CTCTCTCACATCCCAAATGTCTAC	57.5	45.8	24

### 3.2 Specificity of the LAMP assay

**Fig. 2:**
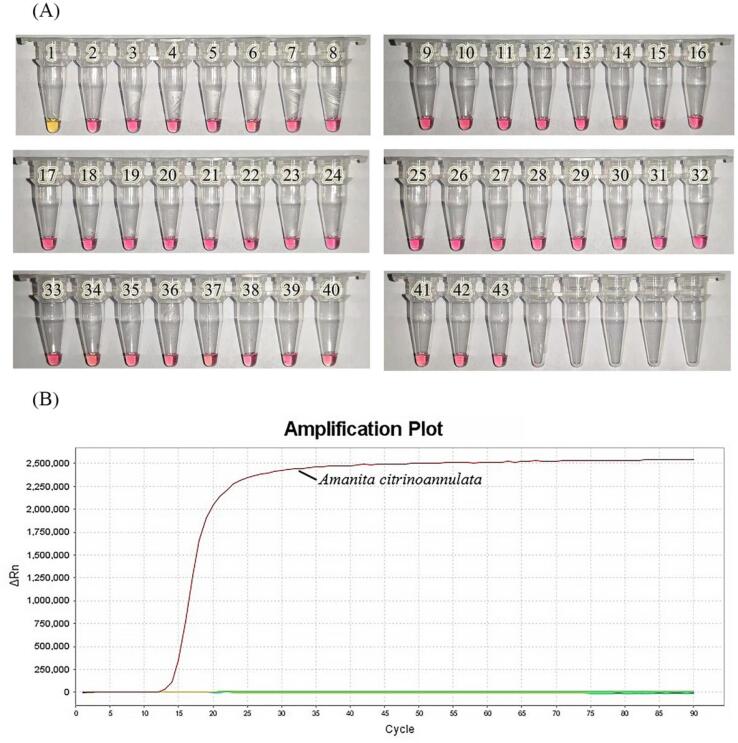
(A)Specific detection using colorimetric LAMP. Tubes 1–42 represent the sample DNA from the following species: 1, *Amanita citrinoannulata*; 2, *Amanita sepiacea*; 3, *Amanita griseofolia*; 4, *Amanita hemibapha*; 5, *Amanita parvipantherina*; 6, *Amanita pseudovaginata*; 7, *Amanita concentrica*; 8, *Amanita spissacea*; 9, *Chlorophyllum molybdites*; 10, *Leucoagaricus rubrotinctus*; 11, *Macrolepiota dolichaula*; 12, *Boletus kauffmanii*; 13, *Butyriboletus yicibus*; 14, *Suillus bovinus*; 15, *Tylopilus neofelleus*; 16, *Tylopilus microsporus*; 17, *Hydnellum caeruleum*; 18, *Hydnellum concrescens*; 19, *Inocybe mixtilis*; 20, *Inocybe rimosa*; 21, *Hypsizygus marmoreus*; 22, *Gymnopus subnudus*; 23, Pleurotus eryngii; 24, *Pleurotus* ostreatus; 25, *Panaeolus subbalteatus*; 26, *Lactarius subbrevipes*; 27, *Russula crustosa*; 28, *Russula rosacea*; 29, *Russula sanguinea*; 30, *Russula senecis*; 31, *Russula* variata; 32, *Russula velenovskyi*; 33, *Laccaria aurantia*; 34, *Flammulina filiformis*; 35, *Lentinula edodes*; 36, *Rhizocybe alba*; 37, *Tricholoma albobrunneum*; 38, *Tricholoma matsutake*; 39, *Tricholoma imbricatum*; 40, *Tricholoma saponaceum*; 41, *Pleurotus* ostreatus; and 42, *Hebeloma crustuliniforme*. Tube 43 was used as the negative control and contained ddH_2_O. (B)Specific detection using real-time fluorescence LAMP. Only positive amplification.

Sequences of common wild mushrooms, poisonous mushrooms, and popular edible fungi, including species of *Amanita*, were used as templates, and ddH_2_O was used as the negative control for the LAMP reaction. The results after amplification at 65 °C for 90 min are shown in Fig. 2. Both colorimetry and real-time LAMP could accurately identify the target species. The positive sample showed color change after 40 min, while the negative sample did not change color until 90 min. The color change was only observed in the *A. citrinoannulata* sample. Other species maintained the same initial color as the negative control, and the primer did not cause false positive amplification of non-target species. This result can also be clearly observed in the results from real-time fluorescence detection. Positive samples were detected at 15 min up to 90 min from the start of amplification, and false-positive results were not observed.

### 3.3 Sensitivity of the LAMP assay

**Fig. 3:**
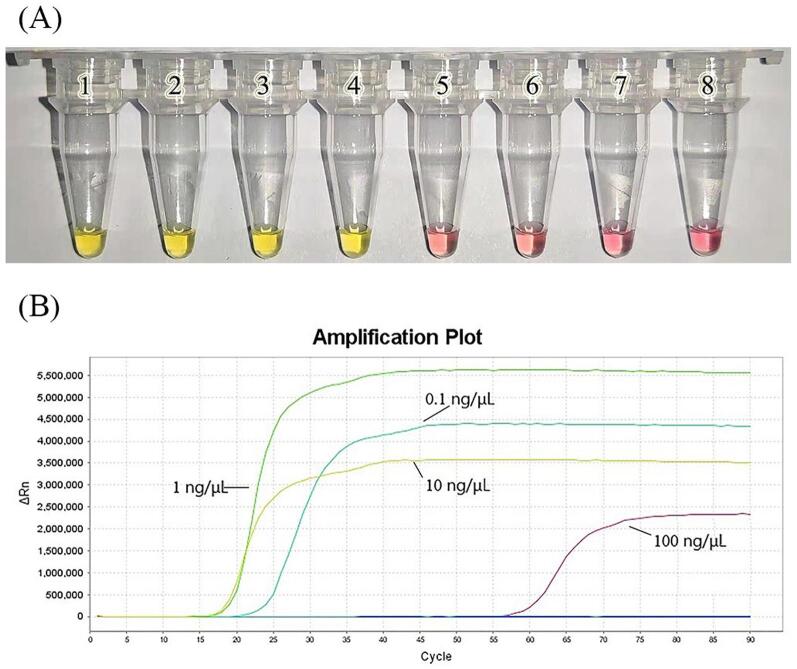
(A) Colorimetric LAMP using serial dilutions of *Amanita citrinoannulata* DNA. 1, 100 ng/μL; 2, 10 ng/μL; 3, 1 ng/μL; 4, 0.1 ng/μL; 5, 0.01 ng/μL; 6, 1 pg/μL; 7, 0.1 pg/μL; 8, ddH_2_O (no-template control). (B)Real-time fluorescence LAMP using serial dilutions of Amanita citrinoannulata DNA.

To determine the minimum amount of sample DNA detectable by these methods, visual LAMP detection and real-time fluorescence LAMP detection were performed with prepared DNA of different concentrations. According to the color change of the tube and the line in the result figure, the limit of detection (LOD) of both new methods were 0.1 ng/μL (Fig. 3). As the reaction mixture contained 2 μL DNA template, the LOD was 0.2 ng. As shown in Fig. 3B, the DNA template with a high concentration of 100 ng / μL after DNA amplification showed the longest reaction time. This is also evidenced by the time required for color change in visual experiments. This may be due to the inhibition of LAMP amplification caused by excessive DNA concentration as well as the high concentration of inhibitors associated with DNA extraction.

### 3.4 Applicability to cooked and digested samples

**Fig. 4:**
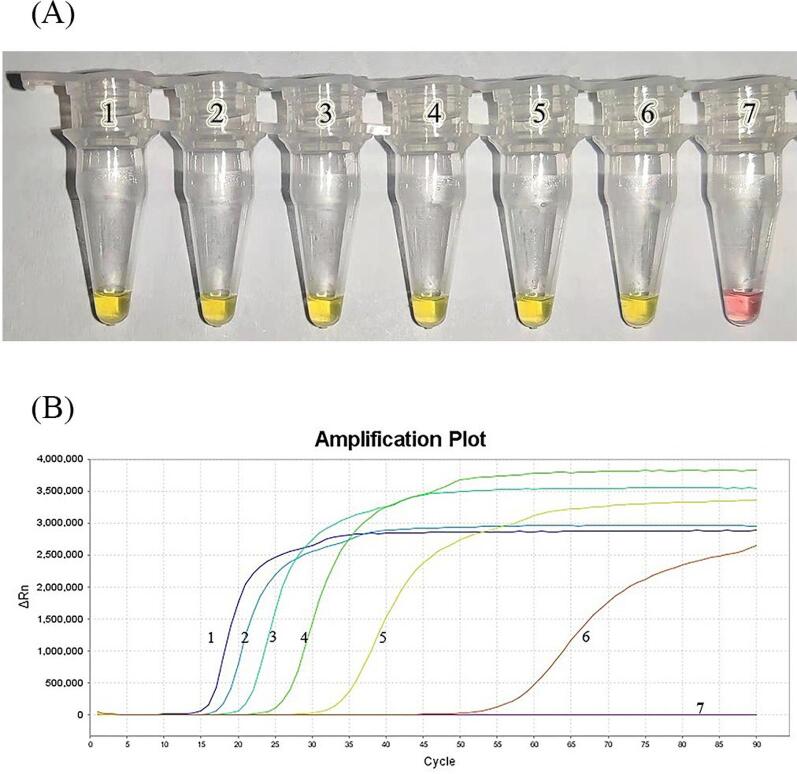
The order of DNA samples is: 1. Boiled and mixed (*Chlorophyllum molybdites*: *Lentinula edodes*: *Amanita citrinoannulata*; same order throughout) 54:45:1; 2. Treated with boiling water and mixed 54:45:1, gastric juice treatment; 3. Treated with boiling water and mixed 25:25:50; 4. Boiled and mixed 25:25:50, gastric juice treatment; 5. Treated with boiling water 1:1:98; 6. Boiled and mixed 1:1:98, gastric juice treatment; and 7. ddH_2_O. (A) Use of colorimetric LAMP products to analyze the DNA extracted from samples treated with boiling water or boiling water and in vitro digestion procedures. (B)Applicability results of real-time LAMP.

In daily life, Chinese people often consume mushroom ‘hotpots’, containing a variety of mushrooms. Therefore, in practical applications, the samples are usually mixed and processed. Furthermore, when mushrooms are eaten by mistake and are digested in the stomach, DNA gets degraded. Our study aimed to evaluate whether the new methods could detect *A. citrinoannulata* in processed sources such as hotpots. To evaluate the feasibility of our method, a LAMP assay was performed using boiling and digested sample mixture. DNA was successfully amplified in both groups by colorimetric LAMP (Fig. 4). The results from real-time fluorescence LAMP analysis also confirmed amplification in the *A. citrinoannulata* (Fig. 4).

## 4 Discussion

LAMP of DNA only requires the implementation of constant temperature conditions without the use of complex instruments. These specific requirements make the new method a relatively simple one that can be used in the absence of specialized equipment. The sensitivity of LAMP is generally 10 times higher than that of PCR (Mashooq et al., 2016). Therefore, LAMP detection methods can replace PCR-based food safety detection methods to some extent in the food industry (Lee, 2017).

The ITS sequences of 93 *Amanita* species were obtained from NCBI, including many *A. citrinoannulata* related species. All primers used in the study were designed based on the sequences of all *A. citrinoannulata* related species. One of the major difficulties in establishing a LAMP detection method is the design of LAMP primers highly specific for certain species (Tomita et al., 2008). DNA barcodes commonly used for fungal identification include ribosomal ITS, large ribosomal subunit gene (LSU), the second largest subunit of RNA polymerase II (RPB2), and β-tubulin (Cai et al., 2012, He et al., 2018, Schoch et al., 2012, Zervakis et al., 2019). Deng et al. (2013) used LSU and ITS gene sequences to identify *Amanita* collected from the Guangdong Province, China. The highly variable regions of ITS ribosomal DNA show interspecies genetic variations and have become important molecular markers for intraspecific phylogenetic development, interspecies variation, and genetic diversity analysis (Bunyard et al., 1996, Dunham et al., 2003, Smith and Sivasithamparam, 2000). Therefore, the ITS gene fragment was selected as the target gene for LAMP design in this study.

However, because the gene sequences of *A. spissa*, *A. spissacea*, and *A. citrinoindusiata* are very similar to those of *A. citrinoannulata*, the primer binding sites designed initially using GLAPD had too few mismatched bases and are prone to produce false positives. Therefore, it was necessary to manually select binding sites, such that only the primers for the most mismatched loci are generated by GLAPD. This ensured that the primer binding sites had sufficient consistency among the same species and sufficient mismatch among different species. At the same time, these primers also met the basic requirements of low GC content and less dimer formation.

After several sensitivity experiments, it was evident that the LAMP reaction was inhibited when the concentration of DNA was 100 ng/μL. This is consistent with the research findings of Njiru et al. (Njiru et al., 2008), and may be due to a fact that mushroom fruiting bodies contain more polysaccharides and phenolic compounds. These secondary metabolites easily affect the DNA amplification results (Abubakar et al., 2021). The amplification efficiency could be improved by constantly purifying the DNA to be used in the experiment. However, DNA dilution can also achieve the purpose of not affecting the efficiency of amplification, and therefore, the concentration of DNA used for the amplification should be noted during application.

A variety of methods have been developed to detect LAMP results, such as turbidity detection (Mori et al., 2001), fluorescence detection (Saull et al., 2016, Spielmann et al., 2019), and gel electrophoresis assays (Sheu et al., 2020). The colorimetric method used in this study is the simplest and most cost-effective method. Compared with the results obtained using the turbidity, HNB, and calcein detection methods, those obtained using the colorimetric method, i.e., the pink and yellow color contrast, are more discernable and obvious. The results of the real-time fluorescence LAMP analysis can give semi-quantitative results. The two detection methods established in this study could be used to observe the results without opening the lid of the centrifuge tube after the reaction. Compared with the electrophoresis detection method, the possibility of aerosol contamination is reduced. Our results show that the rapid DNA-based detection method of *A. citrinoannulata* is useful and does not require examination of sample morphology. Samples containing even small amounts of DNA can be used for the LAMP method described in this study.

This study also has certain limitations. First, the sensitivity is not high enough. In this study, multiple sets of LAMP primers were designed. Finally, in order to ensure the specificity of the sample, primers with relatively low sensitivity were selected. However, these primers could meet the requirements of practical application.

In addition, there are many different species of poisonous mushrooms which show similar appearances. In the case of mushroom poisoning, more than one species of mushrooms might be involved, which implies that a method that identifies multiple species of poisonous mushrooms or a multiplex detection method are necessary. In this study, only a method for *A. citrinoannulata* detection was developed using a real time fluorescent LAMP method, which showed good specificity, sensitivity and promising application potential in different processed or digested samples. In order to detect more species of poisonous mushrooms, this method could be combined with microfluidic chip technology, thereby performing a multiplex mushroom species identification in one reaction. We are collecting and identifying various species of mushrooms from different regions of China and are in the process of preparing a microfluidic chip based multiplex-LAMP method for poison mushrooms identification.

## 5 Conclusion

This is the first study to develop a LAMP method for the specific, sensitive, and rapid identification of *A. citrinoannulata*. Using the new colorimetric method described in this study, results can be obtained in a one-step readout, and the method, therefore, can be used to develop a kit for the on-site detection of *A. citrinoannulata*. Real-time fluorescence LAMP can be used for semi-quantitative detection of *A. citrinoannulata*. In particular, this study demonstrates the possibility of rapid and visual species identification, providing a convenient, accurate, field-adaptable, and low-cost tool for identifying *A. citrinoannulata* in a resource-limited environment. The advantages provided by the LAMP technology could contribute to large-scale screening and industrial quality control in many fields.

## Funding

This work was supported by the National Key R&D Program of China [grant number 2019YFC1604700].

## Declaration of Competing Interest

The authors declare that they have no known competing financial interests or personal relationships that could have appeared to influence the work reported in this paper.
